# Running from Death: Can Fitness Outpace Alcohol’s Harm? Changes in Alcohol Intake, Fitness and All-Cause Mortality in the HUNT Study, Norway

**DOI:** 10.1007/s40279-025-02360-w

**Published:** 2025-12-09

**Authors:** Javaid Nauman, Emma M. L. Ingeström, Atefe R. Tari, Ulrik Wisløff

**Affiliations:** 1 Department of Circulation and Medical Imaging, Faculty of Medicine and Health Sciences, Norwegian University of Science and Technology, Trondheim, Norway; 2 Institute of Public Health, College of Medicine and Health Sciences, United Arab Emirates University, Al-Ain, United Arab Emirates; 3 Department of Neurology, St. Olavs Hospital, Trondheim University Hospital, Trondheim, Norway; 4 Centre for Research on Exercise, Physical Activity and Health, School of Human Movement and Nutrition Sciences, The University of Queensland, Brisbane, QLD Australia

## Abstract

**Background:**

There is no safe lower limit for alcohol intake, and even small amounts increase the risk of premature mortality. It is not known whether a change in cardiorespiratory fitness can modify the association between a change in alcohol intake and mortality.

**Methods:**

We analysed data of the healthy adults from the second (HUNT2; 1995–7) and third (HUNT3; 2006–8) surveys of the population-based Trøndelag Health Study, Norway. Alcohol intake at HUNT2 and HUNT3 was divided into three groups: abstainers, within recommendations (≤ 140 g/week for men, ≤ 70 g/week for women) or above recommendations (> 140 g/week for men, > 70 g/week for women). Using a validated non-exercise prediction equation, we classified participants into two sex- and age-specific fitness groups (unfit: 20% least fit; fit: 80% most fit) at both HUNT2 and HUNT3. Using multi-variable-adjusted Cox analyses, adjusted hazard ratios (aHRs) and 95% confidence intervals (CIs) were estimated for an association between all-cause mortality and a change in alcohol and fitness status.

**Results:**

A total of 24,853 healthy adults (mean [standard deviation] age, 54.7 [12] years; 54.1% women) were included. Over a median follow-up of 16.6 (interquartile range, 16.2–17.1) years, 3921 participants died. Increased alcohol intake from HUNT2 to HUNT3 was associated with an increased risk of mortality. Alcohol abstainers who reported to drink within the recommendations 10 years later (aHR, 1.20; 95% CI 1.00–1.44), and drinkers who increased their intake from within the recommendations at HUNT2 to above at HUNT3 (aHR, 1.25; 95% CI 0.99–1.57) had an increased risk of mortality, compared with the persistent abstainers. Participants drinking within the recommendations at HUNT2 but abstained from drinking at HUNT3 were not at a higher risk of mortality (aHR, 1.14; 95% CI 0.80–1.62). A change in fitness modified the relationship between alcohol intake and all-cause mortality (*P* = 0.03), and participants who remained unfit had higher mortality risks. Compared with the reference group who abstained from alcohol and remained fit from HUNT2 to HUNT3, those who *remained unfit* and persistently abstained, started drinking or consistently drank alcohol within the recommended limits had aHRs of 1.65 (95% CI 1.19–2.30), 1.46 (95% CI 1.04–2.06) and 1.68 (95% CI 1.36–2.08), respectively. For participants who *remained fit*, the mortality risk associated with changes in alcohol intake was not higher than for the reference group, except for those who started drinking [aHR, 1.32 (195% CI 04–1.68)]. Compared with peers remaining fit, decreasing fitness increased the mortality risk among persistent abstainers and consistent drinkers.

**Conclusions:**

Increased alcohol intake over the years was associated with an increased risk of mortality. A change in cardiorespiratory fitness was a better predictor of mortality, and maintaining fitness above the lowest 20% for one’s age and sex attenuated the association between a change in alcohol intake and all-cause mortality.

**Supplementary Information:**

The online version contains supplementary material available at 10.1007/s40279-025-02360-w.

## Key Points

**Table:** 

There is no safe lower limit for alcohol intake; even small amounts increase the risk of premature mortality.
Some evidence suggests that regular physical activity modifies the association between alcohol intake and all-cause mortality; however, it is not known whether a change in cardiorespiratory fitness can modify the association between a change in alcohol intake and mortality.
Our study suggests that maintaining cardiorespiratory fitness above the lowest 20% for one’s age and sex lowers the mortality risk associated with alcohol intake, and a 10-year change in fitness is a better predictor of mortality than contemporary changes in alcohol intake.

## Introduction

Alcohol drinking is customary and well integrated in our culture, with high prevalence and consumption rates across the globe [1–3]. It is commonly believed that a moderate alcohol intake is safe or even health promoting, and previous research may have supported that idea [4]. However, more recent findings show that even small amounts of alcohol increase the risk of several health outcomes, such as cancer, cardiovascular disease and premature death [1, 2]. Last year, the Norwegian Institute of Public Health commented on similar findings [5], and concluded that we should not drink alcohol for health’s sake. The Norwegian Directorate of Health’s recommendations are under revision, but currently states that the alcohol intake should be limited, and not exceed 20 and 10 g/day for men and women, respectively [6, 7]. In contrast, the widely debated Nordic Nutrition Recommendations 2023 state that no safe lower limit for alcohol intake has been established and recommends abstinence [7, 8]. Despite campaigns and other efforts to reduce alcohol intake in the general adult population, the consumption remains stable and a major concern for public health [1], and thus alternative approaches to reduce the risks of alcohol drinking are warranted.

Alcohol consumption influences cardiovascular risk through a complex interplay of biological mechanisms that are often dose dependent. Key pathways include alcohol metabolism-induced oxidative stress, systemic inflammation, endothelial dysfunction, and adverse effects on lipid metabolism and thrombogenicity [9–11]. These processes contribute to the development of atherosclerosis, hypertension and myocardial injury, thereby elevating the risk of cardiovascular disease and mortality [9].

It is well established that regular physical activity and high cardiorespiratory fitness (CRF) are associated with better health and reduced all-cause mortality [12–20]. However, few studies have assessed the joint effects of alcohol intake and physical activity on all-cause mortality [21–24], and none of the previous studies has accounted for temporal changes in alcohol intake or CRF.

Single timepoint assessments may misclassify exposure and underestimate risk associated with drinking behaviour and CRF, which can fluctuate over time [18, 25]. Analysing changes over time is therefore critical to accurately capture their dynamic long-term health effects. In this prospective cohort study, we aimed to examine whether changes in alcohol intake and estimated CRF are associated with the risk of all-cause mortality among Norwegian adults (aged ≥ 20 years). We hypothesised that maintaining fitness above the lowest 20% for one’s age and sex is associated with a reduced mortality risk, regardless of changes in alcohol intake.

## Methods

### Study Design and Population

The Trøndelag Health (HUNT) Study is an extravagant population-based health survey in the county of Trøndelag in central Norway. Four surveys of the HUNT Study have been conducted so far, each just over a decade apart: HUNT1 (1984–6), HUNT2 (1995–7), HUNT3 (2006–8) and HUNT4 (2017–19). All residents (aged ≥ 20 years) in the northern region of Trøndelag received an invitation to each survey. Each visit involved participants filling out extensive questionnaires about their health and lifestyle, as well as a battery of health assessments. Comprehensive details about the HUNT surveys are available in previous publications [26, 27]. In this study, we used data from HUNT2 (August 1995 to June 1997) and HUNT3 (October 2006 to June 2008). This 10-year interval was chosen to establish a contemporary and clearly defined period for assessing changes in exposure variables. Using an additional earlier wave would have required more complex trajectory modelling, and introduced methodological challenges. Out of the 37,069 participants in both HUNT2 and HUNT3, we excluded those with a self-reported history of myocardial infarction, angina, stroke, other cardiovascular diseases or cancer (*n* = 6641). We also excluded those with missing data on alcohol intake (*n* = 1233) and CRF (*n* = 2778), and those with incomplete information on pre-selected potential confounders (*n* = 1564). This left us with a cohort of 24,853 adults (13,435 women and 11,418 men) for an in-depth analysis (sFig. 1 of the Electronic Supplementary Material [ESM]). All participants were informed orally and in writing, and gave a signed consent to partake in the study. The study was approved by the Regional Committee on Medical and Health Research Ethics of Norway (REK 745308).

### Alcohol Intake

We classified participants based on their self- reported abstinence or alcohol intake over 2 weeks. Total quantity in grams of pure alcohol per week was calculated by summing the units of beer [33 cl (4.5%) = 11.9 g], wine [15 cl (12%) = 14.4 g] and spirits/liquor [4 cl (40%) = 12.8 g]. Participants were divided into three groups based on their alcohol intake at HUNT2 and HUNT3: abstainers, within recommendations (≤ 140 g/week for men, ≤ 70 g/week for women) or above recommendations (> 140 g/week for men, > 70 g/week for women) [28].

### Cardiorespiratory Fitness (CRF)

We used a validated non-exercise prediction equation to estimate CRF [29–31], and classified the participants into two sex-specific fitness groups based on age at both HUNT2 and HUNT3. The 20% least fit within each 10-year age category were classified as unfit, whereas the 80% most fit were classified as being fit for their age, as previously described [32]. The fit group represents a broad spectrum of participants above the lowest 20% of age- and sex-specific CRF, rather than exclusively those with high CRF values. The CRF prediction equation was developed in the HUNT Study population and was based on age, waist circumference, resting heart rate and physical activity. The HUNT estimated CRF model has been cross-validated against directly measured peak oxygen uptake [29–31], and its performance is similar or superior compared to other non-exercise prediction equations [31].

### All-Cause Mortality

All participants were followed up on their status (resident/emigrated/dead) in the Norwegian National Population Register. Personnel at the HUNT Research Centre merged HUNT and registry data using the participants’ unique 11-digit personal identification number. We defined the participation date in HUNT3 as the baseline for the follow-up, and followed the participants until the date of death, censoring (emigration) or end of the follow-up of 15 June, 2024, whichever came first.

### Statistical Analyses

We used Cox proportional hazard regression models to examine the association between a change in alcohol intake and all-cause mortality. We grouped participants into nine categories based on their change in alcohol intake: abstainers at both HUNT2 and HUNT3; abstainers at HUNT2 and within recommendations at HUNT3; abstainers at HUNT2 and above recommendations at HUNT3; within recommendations at HUNT2 and abstainers at HUNT3; within recommendations at both HUNT2 and HUNT3; within recommendations at HUNT2 and above recommendations at HUNT3, above recommendations at HUNT2 and abstainers at HUNT3; above recommendations at HUNT2 and within recommendations at HUNT3; or above recommendations at both HUNT2 and HUNT3. In addition, we classified the participants into four categories of CRF change: unfit (age- and sex-specific 20% least fit) at both HUNT2 and HUNT3; unfit at HUNT2 and fit (age- and sex-specific 80% most fit) at HUNT3; fit at HUNT2 and unfit at HUNT3; and fit at both HUNT2 and HUNT3. The survival analyses were conducted using different covariates from HUNT3 in three models. The (1) basic models were stratified by sex and adjusted for attained age. The (2) multivariable models were further adjusted for: body mass index (< 18.5, 18.5–24.9, 25–29.9 or ≥ 30 kg/m^2^); smoking status (never, former, current or occasional); hypertension [yes (systolic blood pressure ≥ 140 mmHg or diastolic blood pressure ≥ 90 mmHg or taking blood pressure medications), or no]; diabetes [yes (self-reported history of diabetes or non-fasting serum glucose > 11.1 mmol/L), or no]; elevated serum total cholesterol [yes (age-based serum cholesterol levels: > 6.1 mmol/L for those < 30 years, > 6.9 mmol/L for those between the ages of 30 and 49 years, > 7.8 mmol/L for those ≥ 50 years), or no]; marital status (married, un-married, divorced/separated or widow/widower); self-reported health status (poor, not so good, good or very good). The last (3) multivariable models were adjusted for the same covariates as the second, with the addition of CRF.

To assess the joint association between a change in alcohol intake and a change in CRF, we derived a combined variable with 36 groups (9 alcohol intake × 4 CRF categories), and repeated the multivariable adjusted analyses. In a separate analysis, we combined the within recommendations and above recommendations in one category of alcohol drinkers, and conducted the joint association analyses using 16 groups (4 alcohol × 4 CRF). In another analysis, we calculated the continuous change in CRF as the difference between HUNT3 and HUNT2 values. This continuous change variable was then categorised into tertiles, representing groups with decreased, stable and increased CRF over the approximately 10-year period. We subsequently created a combined variable of CRF change tertiles and alcohol intake change to examine their joint association with mortality. The proportional hazard assumption was examined by evaluating interaction with time and log-time, and stratified Cox regression conditioning on sex were used, without modelling time-dependent effects. Baseline characteristics are summarised and presented as averages or percentages; results are presented as adjusted hazard ratios (aHRs) with associated 95% confidence intervals (CIs).

In a separate analysis, we divided participants based on meeting or not meeting the physical activity recommendations (weekly 150 min of moderate intensity or 75 min of vigorous activity or a combination of both), and created a change variable for physical activity between HUNT2 and HUNT3. The physical activity change variable was then combined with the alcohol change variable to assess the joint association with all-cause mortality.

We also conducted sensitivity analyses to assess the robustness of our findings by excluding the first 3 years of follow-up to minimise the likelihood of bias due to reverse causality. The statistical analyses were performed using Stata for Windows (Version 16; StataCorp LLC, TX, USA), and all tests were two-sided, with *p* < 0.05 considered significant.

## Results

Characteristics of the participants by the level of alcohol intake at HUNT 3 are presented in Table 1. Alcohol abstainers were older, had a higher prevalence of obesity, diabetes and hypertension, and were more likely to be categorised as unfit compared with alcohol drinkers. However, abstainers had a lower prevalence of hyperlipidaemia and current smoking. Participants’ characteristics by change in alcohol status between HUNT2 and HUNT 3 are presented in sTable 1 of the ESM. Those who increased their alcohol intake over the years were younger, and had a higher prevalence of current smoking. We compared the 24,853 participants who were included with the 12,216 participants who were excluded from the study analysis (sTable 2 of the ESM). The excluded participants were older, and have a worse health profile (high proportion of hypertension, diabetes and poor/not so good general health status) compared with those who were included in the study (sTable2 of the ESM).

**Table 1:** Characteristics of study participants at HUNT 3 by level of alcohol consumption

	Abstainers (*n* = 832)		Withing recommendations (*n* = 22,859)	Above recommendation (*n* = 1162)
Age, mean (SD), years	62.9 (13.2)		54.4 (12.0)	54.6 (9.9)
Female sex, *n* (%)	588 (70.7)		11,954 (52.3)	893 (76.8)
Body mass index, kg/m^2^, *n* (%)				
< 18.5	4 (0.5)		84 (0.4)	6 (0.5)
18.5–24.9	244 (29.3)		6929 (30.3)	425 (36.6)
25.0–29.9	358 (43.0)		10,765 (47.1)	560 (48.2)
≥ 30.0	226 (27.2)		5081 (22.2)	171 (14.7)
Hypertension status, *n* (%)				
No	376 (45.2)		13,877 (60.7)	702 (60.4)
Yes	456 (54.8)		8982 (39.3)	460 (39.6)
Hyperlipidaemia status, *n* (%)				
No		805 (96.7)	21,897 (95.8)	1123 (96.6)
Yes		27 (3.3)	962 (4.2)	39 (3.4)
Smoking status, *n* (%)				
Never	692 (83.2)		9741 (42.6)	299 (25.7)
Former	89 (10.7)		7708 (33.7)	498 (42.9)
Current	41 (4.9)		3934 (17.2)	274 (23.6)
Occasionally	10 (1.2)		1476 (6.5)	91 (7.8)
Diabetes status, *n* (%)				
No	762 (91.6)		21,933 (95.9)	1136 (97.8)
Yes	70 (8.4)		926 (4.1)	26 (2.2)
Marital status, *n* (%)				
Married	561 (67.4)		15,337 (67.1)	799 (68.8)
Unmarried	98 (11.8)		3755 (16.4)	143 (12.3)
Divorced/separated	39 (4.7)		2390 (10.5)	167 (14.4)
Widow/widower	134 (16.1)		1377 (6.0)	53 (4.5)
Health status, *n* (%)				
Poor	15 (1.8)		203 (0.9)	7 (0.6)
Not so good	254 (30.5)		4974 (21.7)	232 (20.0)
Good	448 (53.8)		13,959 (61.1)	692 (59.5)
Very good	115 (13.8)		3723 (16.3)	231 (19.9)
Cardiorespiratory fitness, *n* (%)				
Unfit	174 (20.9)		4015 (17.6)	161 (13.9)
Fit	658 (79.1)		18,844 (82.4)	1001 (86.1)

*SD* standard deviation

Alcohol consumption: within recommendations (≤ 140 g/week for men, ≤ 70 g/week for women); or above recommendations (> 140 g/week for men, > 70 g/week for women)

Cardiorespiratory fitness: unfit, age-sex specific 20% least fit; or fit, age-sex specific 80% most fit

**Fig. 1:**
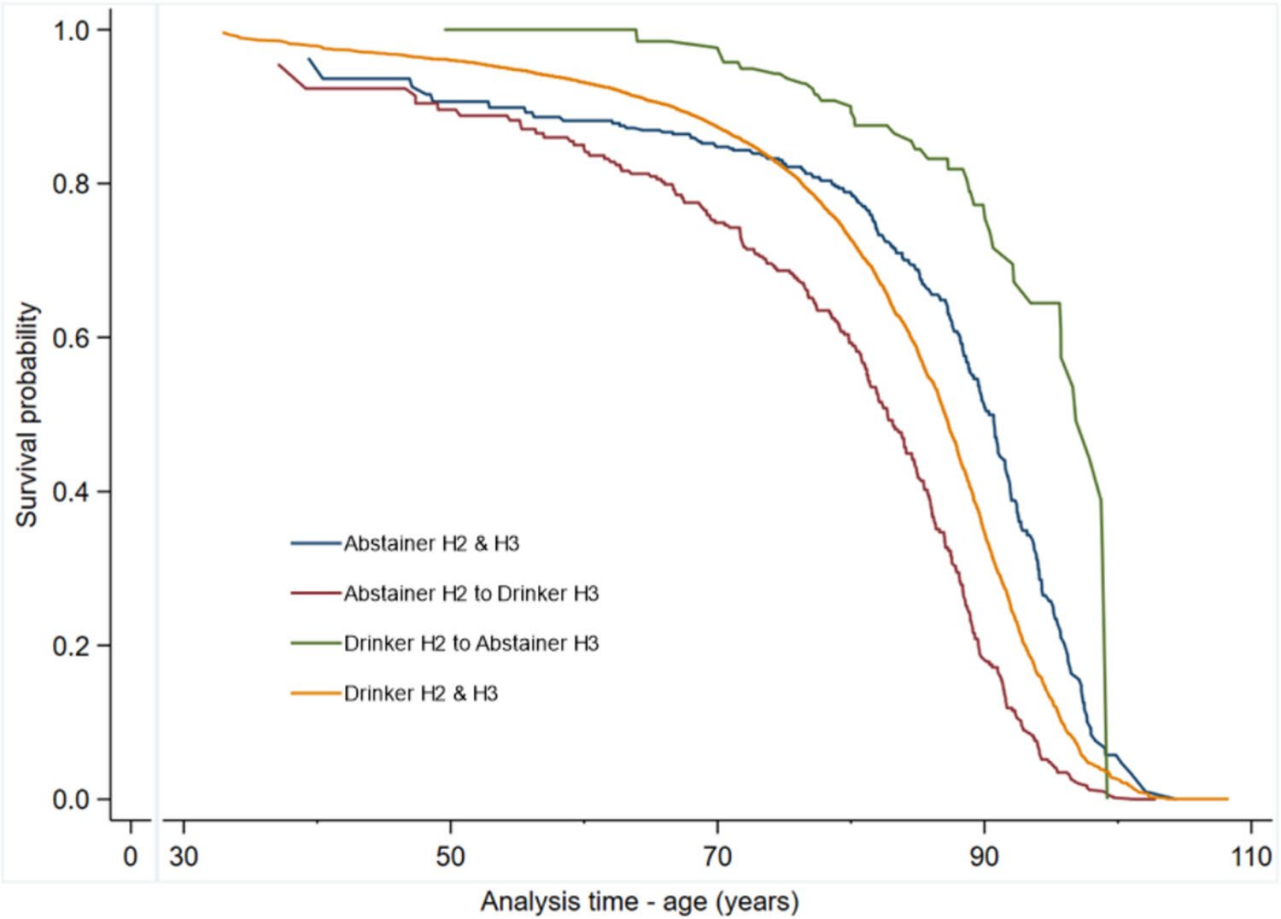
Survival curves associated with a change in alcohol intake. *H2* HUNT2, *H3* HUNT3

During a median follow-up time of 16.6 years (interquartile range, 16.2–17.1 years), a total of 3921 participants died. Compared with the persistent abstinence, the pattern of aHRs suggested that increases in alcohol intake from HUNT2 to HUNT3 may be associated with an increased risk of mortality; however, CIs for several categories were wide, and included the null value (Table 2). For example, alcohol abstainers who reported to drink within the recommendations 10 years later (aHR, 1.20; 95% CI 1.00–1.44), and drinkers who increased their intake from within the recommendations at HUNT2 to above at HUNT3 (aHR, 1.25; 95% CI 0.99–1.57) had increased mortality risks. Participants drinking within the recommendations at the first examination (HUNT2) but abstained from drinking at the second examination (HUNT3) were not at a higher risk of mortality (aHR, 1.14; 95% CI 0.80–1.62) compared to their peers persistently abstaining alcohol. Those who decreased their alcohol from above recommendations at HUNT2 to within at HUNT3 remained at a higher risk (aHR, 1.44; 95% CI 1.06–1.95) [Table 2]. Participants who abstained from alcohol at both HUNT2 and HUNT3 and those who became abstainer at HUNT3 had better survival than those who changed from abstinence at HUNT2 to drink alcohol at HUNT3 and persistently drank alcohol at both HUNT2 and HUNT3 (Fig. 1).

**Table 2:** HRs of all-cause mortality by a change in alcohol status

Alcohol change status^e^	*N*	Deaths	HR (95% CI)^a^	HR (95% CI)^b^	HR (95% CI)^c^
Abstainer HUNT2 and HUNT3	750	204	Reference	Reference	Reference
Abstainer HUNT2, within recommendations HUNT3	945	255	1.27 (1.06–1.53)	1.20 (1.00–1.44)	1.20 (1.00–1.44)
Withing recommendations HUNT2, abstainer HUNT3	82	37	1.34 (0.94–1.90)	1.16 (0.82–1.66)	1.14 (0.80–1.62)
Withing recommendations at HUNT2 and HUNT3	21,610	3216	1.15 (1.00–1.32)	1.06 (0.91–1.23)	1.07 (0.93–1.24)
Withing recommendations HUNT2, above recommendations HUNT3	936	122	1.33 (1.06–1.67)	1.24 (0.98–1.56)	1.25 (0.99–1.57)
Above recommendations HUNT2, within recommendations HUNT3	304	53	1.66 (1.23–2.25)	1.42 (1.05–1.93)	1.44 (1.06–1.95)
Above recommendations at HUNT2 and HUNT3	220	34	1.27 (0.88–1.83)	1.20 (0.83–1.74)	1.22 (0.85–1.77)

*CI* confidence interval, *HR*, hazard ratio

Alcohol consumption categories are based on the average weekly intake in grams of pure alcohol, within recommendations (≤ 140 g/week for men, ≤ 70 g/week for women) or above recommendations (> 140 g/week for men, > 70 g/week for women)

^a^Adjusted for age and sex

^b^Adjusted for age, sex, body mass index, smoking status, hypertension, diabetes, total cholesterol, marital status and general health status

^c^Adjusted for age, sex, body mass index, smoking status, hypertension, diabetes, total cholesterol, marital status, general health status and cardiorespiratory fitness

^e^Alcohol change groups abstainer HUNT2, above recommendations HUNT3, and above recommendations HUNT2, abstainer HUNT3 had zero events

The covariates used in models are from HUNT3 (baseline for the follow-up)

**Fig. 2:**
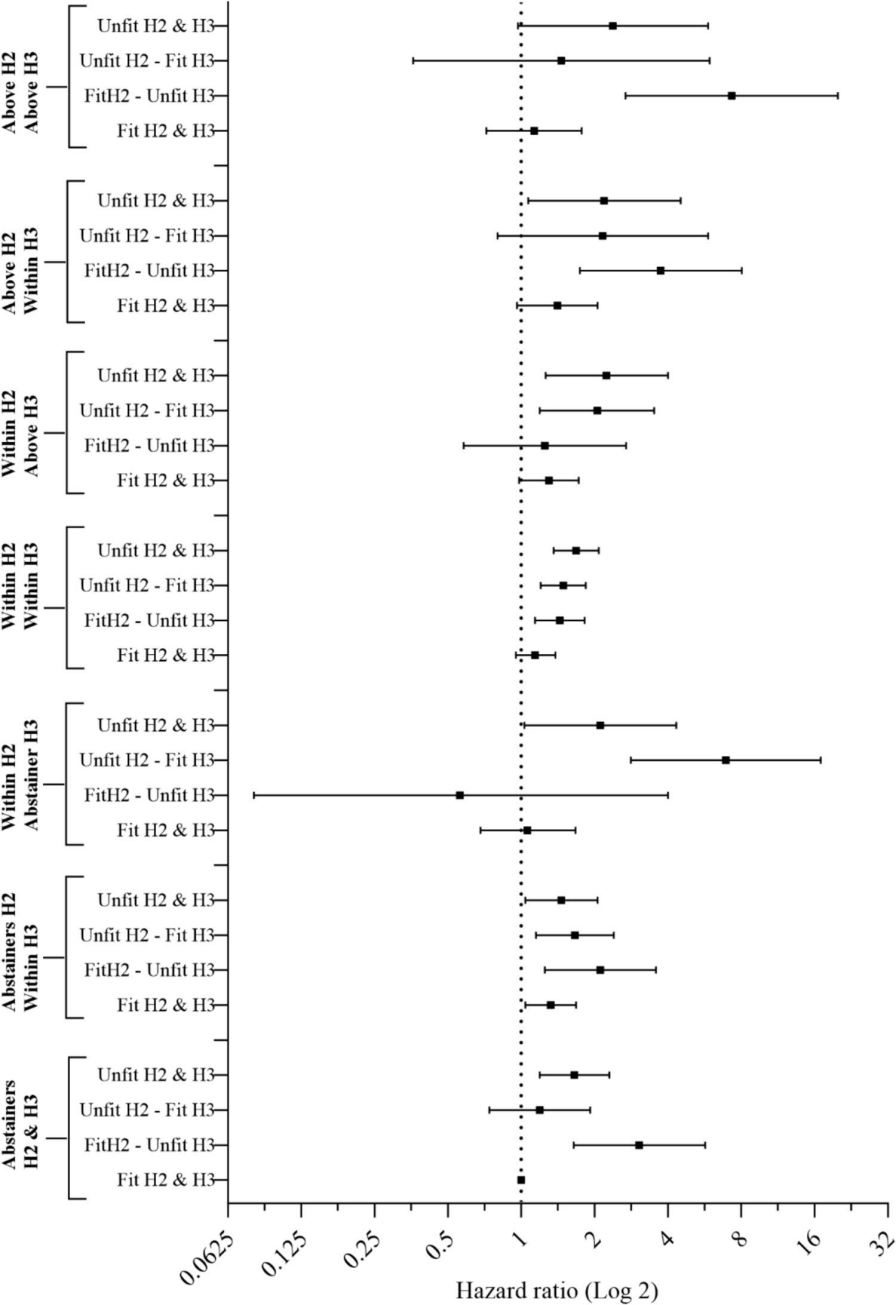
Hazard ratios of all-cause mortality by joint association of a change in alcohol status and a change in cardiorespiratory fitness. Alcohol consumption: within recommendations (≤ 140 g/week for men, ≤ 70 g/week for women); or above recommendations (> 140 g/week for men, > 70 g/week for women). Cardiorespiratory fitness: unfit, age-sex specific 20% least fit; or fit, age-sex specific 80% most fit. Hazard ratios are adjusted for age, sex, body mass index, smoking status, hypertension, diabetes, total cholesterol, marital status and general health status. The covariates used in the model are from HUNT3 (H3) [baseline for follow-up]. *H2* HUNT2

The results of the joint analysis showed that the association between a change in alcohol status and mortality is modified by a change in CRF status (*p*-value of interaction, 0.03) [Fig. 2, sTables 3 and 4 and sFig. 2 of the ESM]. Regardless of the change in the alcohol intake, participants who remained unfit (≤ 20% least fit for one’s age and sex) at both HUNT2 and HUNT3 had an increased risk of mortality. Compared with the reference group, who abstained from alcohol and remained fit (above the lowest 20%) from HUNT2 to HUNT3, those who abstained from alcohol and remained unfit had an increased risk of mortality (aHR, 1.65; 95% CI 1.19–2.30). Similar findings were observed in participants who remained unfit across groups of change in alcohol intake, including those who: changed from abstinence at HUNT2 to drink alcohol within the recommendations at HUNT3 (aHR, 1.46; 95% CI 1.04–2.06); decreased their alcohol intake from within the recommendations at HUNT2 to abstinence at HUNT3 (aHR, 2.11; 95% CI 1.03–4.33); or persistently drank alcohol within the recommendations from HUNT2 to HUNT3 (aHR, 1.68; 95% CI 1.36–2.08). In contrast, participants who remained fit from HUNT2 to HUNT3 did not have a significantly increased risk of mortality across groups of change in alcohol intake compared to the reference group, except those who started drinking and transitioned from abstinence at HUNT2 to drink within the recommendations at HUNT3 (aHR, 1.32; 95% CI 1.04–1.68) [Fig. 2, sTables 3 and 4 of the ESM].

Compared with peers remaining fit, transitioning from *fit to unfit* increased the mortality risk among persistent abstainers (aHR, 3.05; 95% CI 1.64–5.69) and consistent drinkers (aHR, 1.48; 95% CI 1.18–1.87). Participants who transitioned from *unfit to fit* and changed their alcohol intake from being abstainers to drinking and those who consistently drank also had an increased risk of mortality (aHR, 1.67; 95% CI 1.16–2.40, and aHR, 1.51; 95% CI 1.22–1.86, respectively) [sTable 3 of the ESM].

The results of the analysis using tertiles of continuous change in CRF between HUNT2 and HUNT3 showed that the overall pattern was consistent with the main analysis (i.e. using ≤ 20% and above the lowest 20% CRF). However, the associations were attenuated and fewer reached statistical significance after a full adjustment (sTable 5 of the ESM). For example, among participants transitioning from abstinence at HUNT2 to drinking at HUNT3, those in the highest tertile of CRF (i.e. increase) had a higher mortality risk (aHR, 1.43; 95% CI 1.08–1.88) while those with decreased CRF (aHR, 1.30; 95% CI 0.93–1.81) also had an increased mortality risk, compared with the reference group of persistent abstainers who increased their CRF.

The joint analysis of change in alcohol intake and physical activity showed that the pattern of association was similar to that of CRF change, although the aHRs were generally more attenuated, suggesting a weaker protective association compared with the analysis using CRF (sTable 6 of the ESM). Participants who remained below the physical activity recommendations at both HUNT2 and HUNT3 had an increased mortality risk if they began drinking alcohol (aHR, 1.44; 95% CI 1.02–2.02) or were consistent drinkers (aHR, 1.30; 95% CI 0.99–1.70), compared with the reference group of persistent abstainers who met physical activity recommendations. Participants who were abstainers at HUNT2 and changed to drinking at HUNT3, and also transitioned from meeting to not meeting physical activity recommendations, had a particularly increased mortality risk (aHR, 1.58; 95% CI 1.07–2.32). Those who met physical activity recommendations at both timepoints did not show a significantly increased mortality risk across categories of alcohol change (sTable 6 of the ESM). The results of the sensitivity analyses after excluding the first 3 years of follow-up are presented in sTables 7 and 8 of the ESM, and the mortality estimates were not materially changed from the main findings.

## Discussion

In this prospective cohort study of self-reported healthy Norwegian individuals, we found that: (1) changes in alcohol intake over 10 years were associated with a risk of mortality; (2) changes in CRF modified the association between alcohol intake and all-cause mortality; (3) a long-term change in CRF was a better predictor of all-cause mortality than contemporary changes in alcohol intake.

To our knowledge, our study is the first to report the joint association of change in alcohol status and change in CRF with mortality. Previous studies have examined the association between changes in alcohol drinking status and all-cause mortality, and the findings suggest either a J-shaped association [33, 34], or no increase in mortality risk among former non-drinkers increasing their alcohol intake between two examinations [35]. Our results suggest a 20% higher risk of mortality among former abstainers who reported to drink alcohol within the recommendations at the second examination. In addition, the findings highlighted a 44% increased mortality risk among those who reduced their alcohol intake from above to within the recommendations. However, those participants who reduce their alcohol intake from within the recommendations to abstinence were not at an increased risk of mortality than those persistently abstaining from alcohol.

Earlier studies on the joint association between alcohol intake and physical activity relied on assessments from one timepoint, and the results suggest that the positive association between alcohol intake and mortality risk may be diluted by high physical activity levels [22–24]. In a pooled analysis of 36,370 participants from eight British population-based cohorts [23], meeting the current physical activity recommendations (> 7.5 metabolic equivalents of task hour/week) attenuated the association between alcohol intake and mortality risk. The findings were consistent across groups of alcohol consumption, except for participants classified as ‘harmful’ drinkers (UK units of alcohol/week: > 35 women and > 49 men ∼ US units of alcohol/week: > 19.5 women and > 27.5 men). Similar findings were reported by the UK Biobank Study [24], showing that individuals reporting to consume alcohol twice the guidelines or more (≥ 28 UK units of alcohol/week ∼ ≥ 15.8 US units of alcohol/week) had an increased risk of mortality regardless of the physical activity level. Our findings suggest that 10-year changes in CRF may be the better predictor of all-cause mortality than contemporary changes in the alcohol intake. Participants who remained unfit (least 20% of one’s age and sex) at both examinations had an increased risk of mortality regardless of changes in alcohol intake. For instance, persistent abstainers who remained unfit had a 65% increased mortality risk than their peers who remained fit. Additionally, participants who reduced their alcohol drinking from within the recommendations to abstinence, but remained unfit, had an almost a two-fold higher risk of mortality compared with those who persistently abstained from alcohol and remained fit (above the lowest 20%).

To provide a more nuanced perspective on fitness change, we also examined longitudinal changes in CRF using tertiles of continuous change values. The overall pattern was broadly consistent with the binary 20/80 split, with declines in CRF generally associated with a higher mortality risk. However, the hazard ratios were attenuated and many estimates were imprecise, particularly within smaller subgroups. While the 20/80 split provided a clearer risk stratification, the tertile analysis complements the primary findings by highlighting that the direction of change, both decline and improvement, may be relevant for understanding mortality risk trajectories.

Despite the increase in the number of people consuming alcohol worldwide, the proportion of drinkers has not changed significantly since 1990 [3]. The alcohol drinking guidelines have changed over the years, from drinking a low amount of alcohol to zero or very close to zero alcohol consumption for health benefits [3, 6, 7, 10]. Small amounts of drinking alcohol are found to associate with favourable changes in cardiovascular risk factors, such as lipids and glycated haemoglobin [36, 37], translating into a lower risk of diabetes and cardiovascular deaths [1, 2]. However, more recent global estimates show that the slight benefit of cardiovascular disease protection is far outweighed by the health loss from several other diseases associated with any level of alcohol intake [1].

However, increased physical activity and CRF are associated with reduced all-cause and cause-specific mortality, as well as a multitude of health benefits across both sexes and different age categories [13, 16, 20]. The high CRF achieved through incremental physical activity may be the single best predictor of mortality [20]. Our findings represent a significant contribution to the field of public health. These results can be used by clinicians and public health policymakers to emphasise the importance of avoiding very low CRF, and maintaining at least moderate CRF levels specific to age and sex. Furthermore, engagement in physical activity programmes aimed at increasing fitness levels, while concurrently managing alcohol intake has the potential to significantly improve health outcomes and reduce mortality rates.

The main strengths of the study include a relatively large sample size of self-reported healthy participants, a comprehensive source of information on possible confounders at two examinations almost a decade apart, and linkage to national registries ensuring the acquisition of high-quality outcome data. To our knowledge, this is the first study to examine the joint association between a change in alcohol intake and a change in CRF with all-cause mortality. However, there are several limitations that should be considered while interpreting the results. The self-reported data for alcohol drinking may be prone to under-reporting because of social desirability bias, especially for the heavy drinkers who underestimate their alcohol consumption [38]. We classified participants based on alcohol drinking recommendations; however, data on specific drinking patterns such as episodic binge drinking, which have significant health implications [39], were not available. Although, the study sample was relatively healthy after excluding participants with comorbid conditions at baseline, our results may have been influenced by residual unmeasured or unknown variables such as prescribed medications or nutritional data [40], as well as other biological, behavioural and social factors. The CRF was estimated using a non-exercise prediction model, rather than directly measured, which could potentially lead to misclassification. However, the prediction model used to estimate CRF has been cross-validated against the directly measured peak oxygen uptake in the HUNT population [30], and its performance is comparable or superior to other non-exercise models [20, 31]. While direct CRF measurement remains the gold standard, estimating CRF based on non-exercise models represents a validated method in large epidemiological studies [20]. Furthermore, several categories for alcohol change and for the joint alcohol-CRF change analyses, presented in both the main and supplementary materials, were based on a smaller number of events, resulting in wide CIs and limiting the statistical precision and stability of those estimates. Therefore, these particular associations should be interpreted with caution. For the association between alcohol change and mortality, an additional adjustment for CRF (model 3) may introduce a partial over-adjustment because CRF is derived from variables including age, sex, waist circumference, resting heart rate and physical activity. However, the estimates from model 3 were not materially different from those of model 2 without CRF, suggesting that this potential over-adjustment did not meaningfully affect our findings. Our choice of CRF cut-offs into unfit (lowest 20%) and fit (top 80%) groups does not capture potential graded dose–response relationships within the fit group. However, evidence from large cohort studies shows a consistent inverse association between CRF and mortality with no clear upper limit of benefit [19], suggesting that our categorisation does not skew results in favour of CRF benefits or wash out the advantages of very high fitness. Our approach aligns with established epidemiological practice to efficiently identify high-risk individuals [16, 20, 41]. Nonetheless, future studies with larger samples should explore whether very high CRF levels confer additional protection against alcohol-related harms. We studied the long-term temporal changes in alcohol and CRF, and the follow-up of the participants was started at HUNT3 to avoid the ‘*immortal time bias*’ [42], and proportional hazard analyses were stratified by sex, allowing for the baseline hazard function to differ for men and women, which is equivalent to fitting separate Cox hazard models. The HUNT Study population is predominantly Caucasian, and generally presents a more favourable baseline health profile than many other population-based cohorts, and the generalisability of our findings to other cohorts and ethnicities may be limited. Our study was limited to all-cause mortality as an outcome. Future studies with access to cause-specific morbidity data are needed to determine if the protective modifying effect of CRF extends to reducing the risk of serious illness and not just premature death.

## Conclusions

Our findings demonstrated that increased alcohol intake over the years is positively associated with a mortality risk, and avoiding very low CRF (i.e. maintaining CRF above the lowest 20% for one’s age and sex) attenuated this association. Overall, a temporal change in CRF appeared to be a stronger predictor of mortality than contemporary changes in alcohol intake.

## Supplementary Information

Below is the link to the electronic supplementary material.

Supplementary file1 (DOCX 308 KB)

## Declarations

### Availability of Data and Material

Because of privacy policies, data cannot be shared publicly. Researchers associated with Norwegian research institutes can apply for the use of HUNT material: data and samples, given approval by a Regional Committee for Medical and Health Research Ethics. Researchers from other countries are welcome to apply in cooperation with a Norwegian Principal Investigator. Access to the requested HUNT material is given after the application is approved by the HUNT Data Access Committee, and an agreement is signed. The agreement gives the researcher(s) the right to research a specific topic for a limited time period and to publish a decided upon number of articles. More information is available at https://www.ntnu.edu/hunt/research.

### Patient and Public Involvement

We are dependent on the experiences and input of HUNT participants to identify needs, solutions and challenges. Therefore, participants have been involved in all the surveys, from the planning stage to the final evaluation. Many of them have contributed to several HUNT surveys, giving them a unique perspective. We have taken their input seriously, and all feedback has been recorded and further followed up.
