# Supplementary material for: Running from Death: Can Fitness Outpace Alcohol’s Harm? Changes in Alcohol Intake, Fitness and All-Cause Mortality in the HUNT Study, Norway
Source: Sports Med. 2025 Dec 9;56(4):1023–33. doi: 10.1007/s40279-025-02360-w (PMC13124876; doi:10.1007/s40279-025-02360-w)
Supplement: Supplementary file 1 — Supplementary file1 (DOCX 308 KB) [file 40279_2025_2360_MOESM1_ESM.docx]

**Supplementary Materials**

Title: “Running from Death: Can Fitness Outpace Alcohol’s Harm? Changes in Alcohol Intake, Fitness, and All-Cause Mortality in the HUNT Study, Norway.”

Running Head: Fitness, alcohol and mortality

Journal name: Sports Medicine

Authors and affiliations: Javaid Nauman^1,2^, Emma M. L. Ingeström^1^, Atefe R. Tari^1,3^, Ulrik Wisløff^1,4^.

^1^Department of Circulation and Medical Imaging, Faculty of Medicine and Health Sciences, Norwegian University of Science and Technology, Trondheim, Norway.

^2^Institute of Public Health, College of Medicine and Health Sciences, United Arab Emirates University, Al-Ain, United Arab Emirates.

^3^Department of Neurology, St. Olavs Hospital, Trondheim University Hospital, Trondheim, Norway.

^4^Centre for Research on Exercise, Physical Activity and Health, School of Human Movement and Nutrition Sciences, The University of Queensland, Brisbane, Queensland, Australia.

Corresponding author: Javaid Nauman, Email: [javaid.nauman@ntnu.no](mailto:javaid.nauman@ntnu.no); javaid.nauman@uaeu.ac.ae

Content List

**sTable 1**. Characteristics of study participants by change in alcohol consumption……………………………….3

**s Table 2**. Comparison of participants included in the study analyses vs those who were excluded……………...5

**sTable 3**. Hazard ratios of all-cause mortality by joint association of change in alcohol drinking status and change in CRF from HUNT2 (H2) to HUNT3 (H3)………………………………………………………………6

**sTable 4.** Hazard ratios of all-cause mortality by joint association of change in alcohol drinking status and change in CRF from HUNT2 (H2) to HUNT3 (H3)………………………………………………………………7

**sTable 5.** Hazard ratios of all-cause mortality by joint association of change in alcohol drinking status and continuous change in CRF from HUNT2 (H2) to HUNT3 (H3)………………………………………………….9

**sTable 6.** Hazard ratios of all-cause mortality by joint association of change in alcohol drinking status and change in physical activity (PA) from HUNT2 (H2) to HUNT3 (H3)…………………………………………...10

**sTable 7.** Hazard ratios of all-cause mortality by change in alcohol drinking status from HUNT2 (H2) to HUNT3 (H3), after excluding the first 3 years of follow-up……………………………………………………..11

**sTable 8.** Hazard ratios of all-cause mortality by joint association of change in alcohol drinking status and change in CRF from HUNT2 (H2) to HUNT3 (H3), after excluding the first 3 years of follow-up…………….12

**sFigure 1**. Flow chart of study participants……………………………………………………………………....14

**sFigure 2.** Survival curves associated with change in cardiorespiratory fitness according to change in alcohol intake……………………………………………………………………………………………………………...15

**sTable 1. Characteristics of study participants by change in alcohol consumption.**

| **HUNT2** | | **Abstainers** | | | | **Within recommendations** | | | **Above recommendations** | |
| --- | --- | --- | --- | --- | --- | --- | --- | --- | --- | --- |
| **HUNT3** | | **Abstainers (n=750)** | **Within recommendations (n=945)** | | **Above recommendations (n=6)** | **Abstainers (n=82)** | **Within recommendations (n=21,610)** | **Above recommendations (n=936)** | **Within recommendations (n=304)** | **Above recommendations**  **(n=220)** |
| Age, mean (SD), years | | 62.7 (13.1) | | 59.7 (13.4) | 53.8 (10.0) | 65.0 (14.8) | 54.3 (11.8) | 53.9 (9.9) | 50.7 (13.5) | 57.7 (9.0) |
| Female sex, No. (%) | | 519 (69.2) | | 617 (65.3) | 5 (83.3) | 69 (84.2) | 11,512 (51.6) | 721 (77.0) | 185 (60.9) | 167 (75.9) |
| Body mass index, kg/m^2^, No. (%) | | | | |  |  |  |  |  |  |
| <18.5 | 4 (0.5) | | 8 (0.9) | 0 | 0 | 73 (0.3) | 4 (0.4) | 3 (1.0) | 2 (0.9) |  |
| 18.5-24.9 | 217 (28.9) | | 268 (28.4) | 1 (16.7) | 27 (32.9) | 6562 (30.4) | 357 (38.1) | 99 (32.6) | 67 (30.5) |  |
| 25.0-29.9 | 328 (43.7) | | 403 (42.6) | 4 (66.6) | 30 (36.6) | 10,225 (47.3) | 435 (46.5) | 137 (45.1) | 121 (55.0) |  |
| ≥30.0 | 201 (26.8) | | 266 (28.1) | 1 (16.7) | 25 (30.5) | 4750 (22.0) | 140 (15.0) | 65 (21.3) | 30 (13.6) |  |
| Hypertension status, No. (%) | | | |  |  |  |  |  |  |  |
| No | 344 (45.9) | | 479 (50.7) | 2 (33.3) | 32 (39.0) | 13,204 (61.1) | 594 (63.5) | 194 (63.8) | 106 (48.2) |  |
| Yes | 406 (54.1) | | 466 (49.3) | 4 (66.7) | 50 (61.0) | 8406 (38.9) | 342 (36.5) | 110 (36.2) | 114 (51.8) |  |
| Hyperlipidaemia status, No. (%) | | | |  |  |  |  |  |  |  |
|  | No | 726 (96.8) | | 912 (96.5) | 6 (100) | 79 (96.3) | 20,699 (95.8) | 905 (96.7) | 286 (94.1) | 212 (96.4) |
|  | Yes | 24 (3.2) | | 33 (3.5) | 0 | 3 (3.7) | 911 (4.2) | 31 (3.3) | 18 (5.9) | 8 (3.6) |
| Smoking status, No. (%) | | | |  |  |  |  |  |  |  |
|  | Never | 633 (84.4) | | 594 (62.9) | 1 (16.7) | 59 (71.9) | 9056 (41.9) | 242 (25.9) | 91 (29.9) | 56 (25.4) |
|  | Former | 81 (10.8) | | 216 (22.9) | 1 (16.7) | 8 (9.8) | 7383 (34.2) | 394 (42.1) | 109 (35.9) | 103 (46.8) |
|  | Current | 28 (3.7) | | 101 (10.7) | 6 (50.0) | 13 (15.9) | 3754 (17.4) | 224 (23.9) | 79 (26.0) | 47 (21.4) |
|  | Occasionally | 8 (1.1) | | 34 (3.6) | 1 (16.7) | 2 (2.4) | 1417 (6.5) | 76 (8.1) | 25 (8.2) | 14 (6.4) |
| Diabetes status, No. (%) | | | | |  |  |  |  |  |  |
|  | No | 687 (91.6) | | 889 (94.1) | 6 (100) | 75 (91.5) | 20,752 (96.0) | 913 (97.5) | 292 (96.0) | 217 (98.6) |
|  | Yes | 63 (8.4) | | 56 (5.9) | 0 | 7 (835) | 858 (4.0) | 23 (2.5) | 12 (4.0) | 3 (1.4) |
| Marital status, No. (%) | | | | | |  |  |  |  |  |
|  | Married | 515 (68.7) | | 604 (63.9) | 4 (66.6) | 46 (56.1) | 14,570 (67.4) | 637 (68.1) | 163 (53.6) | 158 (71.8) |
|  | Unmarried | 87 (11.6) | | 120 (12.7) | 1 (16.7) | 11 (13.4) | 3547 (16.4) | 120 (12.8) | 88 (29.0) | 22 (10.0) |
|  | Divorced/separated | 37 (4.9) | | 99 (10.5) | 1 (16.7) | 2 (2.4) | 2249 (10.4) | 137 (14.6) | 42 (13.8) | 29 (13.2) |
|  | Widow/widower | 111 (14.8) | | 122 (12.9) | 0 | 23 (28.1) | 1244 (5.8) | 42 (4.5) | 11 (3.6) | 11 (5.0) |

*The table continues on the next page.*

**sTable 1.** Continued.

| Health status, No. (%) | | |  |  |  |  |  |  |  |
| --- | --- | --- | --- | --- | --- | --- | --- | --- | --- |
| Poor | 13 (1.7) | 18 (1.9) | 0 | 2 (2.4) | 181 (0.8) | 3 (0.3) | 4 (1.3) | 4 (1.8) |  |
| Not so good | 226 (30.1) | 301 (31.8) | 1 (16.7) | 28 (34.2) | 4600 (21.3) | 186 (19.9) | 73 (24.0) | 45 (20.5) |  |
| Good | 405 (54.0) | 496 (52.5) | 4 (66.6) | 43 (52.4) | 13,287 (61.5) | 567 (60.6) | 176 (57.9) | 121 (55.0) |  |
| Very good | 106 (14.1) | 130 (13.8) | 1 (16.7) | 9 (11.0) | 3542 (16.4) | 180 (19.2) | 51 (16.8) | 50 (22.7) |  |
| Cardiorespiratory fitness, No. (%) | | | |  |  |  |  |  |  |
|  | Unfit | 156 (20.8) | 202 (21.4) | 1 (16.7) | 18 (21.9) | 3755 (17.4) | 128 (13.7) | 58 (19.1) | 32 (14.5) |
|  | Fit | 594 (79.2) | 743 (78.6) | 5 (83.3) | 64 (78.1) | 17,855 (82.6) | 808 (86.3) | 246 (80.9) | 188 (85.5) |

Alcohol consumption: within recommendations (≤140 g/week for men, ≤70 g/week for women); or above recommendations (>140 g/week for men, >70 g/week for women).

Cardiorespiratory fitness: Age-specific and sex-specific fitness: unfit (≤20% of participants) and fit (>20% of participants).

HUNT, the Trøndelag Health Study.

**sTable 2. Comparison of participants included in the study analyses vs those who were excluded**

|  | | | Included  (n=24,853) | | Excluded*^a^  (n=12,216) |
| --- | --- | --- | --- | --- | --- |
| Age, mean (SD), y | | | 54.7 (12.0) | | 65.2 (12.0) |
| Female sex, No. (%) | | | 13,435 (54.1) | | 7029 (57.5) |
| Body mass index, kg/m^2^, No. (%) | | | | | |
| <18.5 | | 94 (0.4) | | 75 (0.6) |  |
| 18.5-24.9 | | 7598 (30.6) | | 3133 (26.2) |  |
| 25.0-29.9 | | 11,683 (47.0) | | 5419 (45.3) |  |
| ≥30.0 | | 5478 (22.0) | | 3332 (27.9) |  |
| Hypertension status, No. (%) | | | | |  |
| No | | 14,955 (60.2) | | 4678 (38.3) |  |
| Yes | | 9898 (39.8) | | 7537 (61.7) |  |
| Hyperlipidaemia status, No. (%) | | | | |  |
|  | No | | | 23,825 (95.9) | 10,894 (96.2) |
|  | Yes | | | 1028 (4.1) | 427 (3.8) |
| Smoking status, No. (%) | | | | |  |
|  | Never | | 10,732 (43.2) | | 4230 (38.2) |
|  | Former | | 8295 (33.4) | | 4403 (39.8) |
|  | Current | | 4249 (17.1) | | 1766 (16.0) |
|  | Occasionally | | 1577 (6.3) | | 660 (6.0) |
| Diabetes status, No. (%) | | | | | |
|  | No | | 23,831 (95.9) | | 11,187 (91.6) |
|  | Yes | | 1022 (4.1) | | 1023 (8.4) |
| Marital status, No. (%) | | | | |  |
|  | Married | | 16,697 (67.2) | | 7650 (62.7) |
|  | Unmarried | | 3996 (16.1) | | 1237 (10.1) |
|  | Divorced/separated | | 2596 (10.4) | | 1063 (8.7) |
|  | Widow/widower | | 1564 (6.3) | | 2254 (18.5) |
| Health status, No. (%) | | | | |  |
| Poor | 225 (0.9) | | 311 (2.8) |  |  |
| Not so good | 5460 (22.0) | | 4284 (38.9) |  |  |
| Good | 15,099 (60.7) | | 5662 (51.4) |  |  |
| Very good | 4069 (16.3) | | 753 (6.9) |  |  |
| Cardiorespiratory fitness, No. (%) | | | | | |
|  | | Unfit | 4350 (17.5) | | 1894 (19.7) |
|  |  | Fit | 20,503 (82.5) | | 7722 (80.3) |
| *Exclusion was due to history of myocardial infarction, angina pectoris, stroke, other cardiovascular diseases, cancer, missing data on alcohol intake, cardiorespiratory fitness, smoking, cholesterol, health status, BMI or marital status (see the Method section for details).  ^a^Data available: BMI (N=11,959), hypertension (N=12,215), hyperlipidaemia (N=11,321), smoking (N=11,059), diabetes (N=12,210), marital status (N=12,204), health status (N=11,010), cardiorespiratory fitness (N=9616). Cardiorespiratory fitness: unfit, age-sex specific 20% least fit; or fit, age-sex specific 80% most fit. | | | | | |

**sTable 3. Hazard ratios of all-cause mortality by joint association of change in alcohol drinking status and change in CRF from HUNT2 (H2) to HUNT3 (H3).**

|  |  |  | **Model 1** | **Model 2** |
| --- | --- | --- | --- | --- |
|  | **N** | **Deaths** | **HR (95% CI)** | **HR (95% CI)** |
| Abstainer at H2 & H3 – Unfit at H2 & H3 | 119 | 52 | 1.69 (1.22-2.35) | 1.65 (1.19-2.30) |
| Abstainer at H2 & H3 – Unfit at H2 & Fit at H3 | 78 | 20 | 1.27 (0.79-2.05) | 1.19 (0.74-1.92) |
| Abstainer at H2 & H3 – Fit at H2 & Unfit at H3 | 37 | 11 | 2.85 (1.53-5.29) | 3.05 (1.64-5.69) |
| **Abstainer at H2 & H3 – Fit at H2 & Fit at H3** | 516 | 121 | Reference | Reference |
|  |  |  |  |  |
| Abstainer H2, drinker H3 - Unfit H2 & H3 | 137 | 47 | 1.62 (1.16-2.28) | 1.46 (1.04-2.06) |
| Abstainer H2, drinker H3 - Unfit H2 & Fit H3 | 110 | 39 | 1.75 (1.22-2.51) | 1.67 (1.16-2.40) |
| Abstainer H2, drinker H3 - Fit H2 & Unfit H3 | 65 | 16 | 2.36 (1.40-3.99) | 2.11 (1.24-3.57) |
| Abstainer H2, drinker H3 - Fit H2 & Fit H3 | 638 | 153 | 1.38 (1.09-1.76) | 1.31 (1.03-1.67) |
|  |  |  |  |  |
| Drinker H2, Abstainer H3 - Unfit H2 & H3 | 16 | 8 | 2.49 (1.22-5.11) | 2.11 (1.03-4.33) |
| Drinker H2, Abstainer H3 - Unfit H2 & Fit H3 | 8 | 5 | 6.98 (2.85-17.08) | 6.89 (2.81-16.90) |
| Drinker H2, Abstainer H3 - Fit H2 & Unfit H3 | 2 | 1 | 1.13 (0.16-8.12) | 0.56 (0.08-4.01) |
| Drinker H2, Abstainer H3 - Fit H2 & Fit H3 | 56 | 23 | 1.24 (0.79-1.94) | 1.06 (0.68-1.66) |
|  |  |  |  |  |
| Drinker H2, Drinker H3 - Unfit H2 & H3 | 2297 | 488 | 1.97 (1.61-2.41) | 1.71 (1.38-2.11) |
| Drinker H2, Drinker H3 - Unfit H2 & Fit H3 | 2055 | 362 | 1.71 (1.39-2.10) | 1.51 (1.22-1.86) |
| Drinker H2, Drinker H3 - Fit H2 & Unfit H3 | 1676 | 231 | 1.72 (1.38-2.16) | 1.48 (1.18-1.87) |
| Drinker H2, Drinker H3 - Fit H2 & Fit H3 | 17,042 | 2344 | 1.25 (1.04-1.51) | 1.15 (0.95-1.39) |

HUNT, the Trøndelag Health Study; HR, hazard ratios; CI, confidence interval; CRF, cardiorespiratory fitness. Alcohol consumption categories are based on either abstainers or drinking alcohol. CRF: unfit, age-sex specific 20% least fit; or fit, age-sex specific 80% most fit.

Model 1 is adjusted for age and sex.

Model 2 is adjusted for age, sex, body mass index, smoking status, hypertension, diabetes, total cholesterol, marital status, and general health status.

**sTable 4. Hazard ratios of all-cause mortality by joint association of change in alcohol drinking status and change in CRF from HUNT2 (H2) to HUNT3 (H3).**

|  |  |  | **Model 1** | **Model 2** |
| --- | --- | --- | --- | --- |
|  | **N** | **Deaths** | **HR (95% CI)** | **HR (95% CI)** |
| Abstainer at H2 & H3 |  |  |  |  |
| Unfit at H2 & H3 | 119 | 52 | 1.69 (1.22-2.35) | 1.65 (1.19-2.30) |
| Unfit at H2 & Fit at H3 | 78 | 20 | 1.27 (0.79-2.05) | 1.19 (0.74-1.92) |
| Fit at H2 & Unfit at H3 | 37 | 11 | 2.85 (1.53-5.29) | 3.05 (1.64-5.69) |
| **Fit at H2 & Fit at H3** | 516 | 121 | Reference | Reference |
|  |  |  |  |  |
| Abstainer H2, within guidelines H3 |  |  |  |  |
| Unfit at H2 & H3 | 137 | 47 | 1.62 (1.16-2.28) | 1.46 (1.04-2.06) |
| Unfit at H2 & Fit at H3 | 110 | 39 | 1.74 (1.22-2.50) | 1.66 (1.15-2.40) |
| Fit at H2 & Unfit at H3 | 65 | 16 | 2.36 (1.40-3.99) | 2.11 (1.25-3.57) |
| Fit at H2 & Fit at H3 | 633 | 153 | 1.39 (1.09-1.76) | 1.32 (1.04-1.68) |
|  |  |  |  |  |
| Abstainer H2, above guidelines H3 |  |  |  |  |
| Unfit at H2 & H3 | 0 | - | - | - |
| Unfit at H2 & Fit at H3 | 0 | - | - | - |
| Fit at H2 & Unfit at H3 | 1 | 0 | - | - |
| Fit at H2 & Fit at H3 | 5 | 0 | - | - |
|  |  |  |  |  |
| Within guidelines H2, Abstainer H3 |  |  |  |  |
| Unfit at H2 & H3 | 16 | 8 | 2.49 (1.22-5.11) | 2.11 (1.03-4.33) |
| Unfit at H2 & Fit at H3 | 8 | 5 | 6.99 (2.89-17.13) | 6.91 (2.82-16.96) |
| Fit at H2 & Unfit at H3 | 2 | 1 | 1.13 (0.16-8.07) | 0.56 (0.08-4.01) |
| Fit at H2 & Fit at H3 | 56 | 23 | 1.24 (0.79-1.94) | 1.06 (0.68-1.67) |
|  |  |  |  |  |
| Within guidelines H2, within guidelines H3 |  |  |  |  |
| Unfit at H2 & H3 | 2189 | 462 | 1.93 (1.58-2.37) | 1.68 (1.36-2.08) |
| Unfit at H2 & Fit at H3 | 1945 | 341 | 1.68 (1.36-2.07) | 1.49 (1.20-1.84) |
| Fit at H2 & Unfit at H3 | 1566 | 213 | 1.66 (1.32-2.08) | 1.44 (1.14-1.82) |
| Fit at H2 & Fit at H3 | 15,910 | 2200 | 1.24 (1.03-1.50) | 1.14 (0.95-1.38) |
|  |  |  |  |  |
| Within guidelines H2, above guidelines H3 - |  |  |  |  |
| Unfit at H2 & H3 | 63 | 13 | 2.77 (1.56-4.92) | 2.24 (1.26-4.00) |
| Unfit at H2 & Fit at H3 | 79 | 15 | 2.32 (1.36-3.97) | 2.05 (1.19-3.51) |
| Fit at H2 & Unfit at H3 | 65 | 7 | 1.70 (0.79-3.66) | 1.25 (0.58-2.70) |
| Fit at H2 & Fit at H3 | 729 | 87 | 1.43 (1.08-1.89) | 1.30 (0.98-1.72) |

*The table continues on the next page.*

**sTable 4.** Continued.

| Above guidelines H2, Abstainer H3 |  |  |  |  |
| --- | --- | --- | --- | --- |
| Unfit at H2 & H3 | 0 | - | - | - |
| Unfit at H2 & Fit at H3 | 0 | - | - | - |
| Fit at H2 & Unfit at H3 | 0 | - | - | - |
| Fit at H2 & Fit at H3 | 0 | - | - | - |
|  |  |  |  |  |
| Above guidelines H2, within guidelines H3- |  |  |  |  |
| Unfit at H2 & H3 | 27 | 8 | 3.33 (1.63-6.81) | 2.19 (1.07-4.51) |
| Unfit at H2 & Fit at H3 | 22 | 4 | 2.36 (0.87-6.41) | 2.16 (0.80-5.86) |
| Fit at H2 & Unfit at H3 | 31 | 7 | 5.42 (2.53-11.65) | 3.74 (1.74-8.05) |
| Fit at H2 & Fit at H3 | 224 | 34 | 1.60 (1.09-2.34) | 1.41 (0.96-2.06) |
|  |  |  |  |  |
| Above guidelines H2, above guidelines H3 |  |  |  |  |
| Unfit at H2 & H3 | 18 | 5 | 2.95 (1.20-7.22) | 2.38 (0.97-5.85) |
| Unfit at H2 & Fit at H3 | 9 | 2 | 1.83 (0.45-7.42) | 1.46 (0.36-5.93) |
| Fit at H2 & Unfit at H3 | 14 | 4 | 8.77 (3.32-23.85) | 7.31 (2.68-19.93) |
| Fit at H2 & Fit at H3 | 179 | 23 | 1.21 (0.77-1.89) | 1.13 (0.72-1.77) |

HUNT, the Trøndelag Health Study; HR, hazard ratios; CI, confidence interval; CRF, cardiorespiratory fitness. Alcohol consumption categories are based on the average weekly intake in grams of pure alcohol: within recommendations (≤140 g/week for men, ≤70 g/week for women) or above recommendations (>140 g/week for men, >70 g/week for women). CRF: unfit, age-sex specific 20% least fit; or fit, age-sex specific 80% most fit.

Model 1 is adjusted for age and sex.

Model 2 is adjusted for age, sex, body mass index, smoking status, hypertension, diabetes, total cholesterol, marital status, and general health status.

**sTable 5. Hazard ratios of all-cause mortality by joint association of change in alcohol drinking status and continuous change in CRF from HUNT2 (H2) to HUNT3 (H3).**

|  |  |  | **Model 1** | **Model 2** |
| --- | --- | --- | --- | --- |
|  | **N** | **Deaths** | **HR (95% CI)** | **HR (95% CI)** |
| Abstainer at H2 & H3 – CRF decreased | 178 | 48 | 1.30 (0.92-1.84) | 1.27 (0.90-1.81) |
| Abstainer at H2 & H3 – CRF stable | 257 | 64 | 0.99 (0.72-1.37) | 1.05 (0.76-1.44) |
| **Abstainer at H2 & H3 – CRF increased** | 315 | 92 | Reference | Reference |
|  |  |  |  |  |
| Abstainer H2, drinker H3 - CRF decreased | 268 | 57 | 1.39 (1.01-1.94) | 1.30 (0.93-1.81) |
| Abstainer H2, drinker H3 - CRF stable | 313 | 82 | 1.18 (0.88-1.59) | 1.09 (0.81-1.47) |
| Abstainer H2, drinker H3 - CRF increased | 370 | 116 | 1.44 (1.10-1.90) | 1.43 (1.08-1.88) |
|  |  |  |  |  |
| Drinker H2, Abstainer H3 - CRF decreased | 20 | 6 | 1.80 (0.79-4.11) | 1.41 (0.62-3.24) |
| Drinker H2, Abstainer H3 - CRF stable | 27 | 13 | 1.47 (0.82-2.63) | 1.34 (0.75-2.40) |
| Drinker H2, Abstainer H3 - CRF increased | 35 | 18 | 1.28 (0.77-2.13) | 1.13 (0.68-1.89) |
|  |  |  |  |  |
| Drinker H2, Drinker H3 - CRF decreased | 7819 | 1010 | 1.28 (1.03-1.59) | 1.14 (0.91-1.43) |
| Drinker H2, Drinker H3 - CRF stable | 7687 | 1109 | 1.18 (0.59-1.47) | 1.12 (0.90-1.40) |
| Drinker H2, Drinker H3 - CRF increased | 7564 | 1306 | 1.23 (1.00-1.53) | 1.15 (0.93-1.43) |

HUNT, the Trøndelag Health Study; HR, hazard ratios; CI, confidence interval; CRF, cardiorespiratory fitness. Alcohol consumption categories are based on either abstainers or drinking alcohol. CRF values are tertiles (decreased, stable, increased) of continuous change in CRF between HUNT2 and HUNT3.

Model 1 is adjusted for age and sex.

Model 2 is adjusted for age, sex, body mass index, smoking status, hypertension, diabetes, total cholesterol, marital status, and general health status.

**sTable 6. Hazard ratios of all-cause mortality by joint association of change in alcohol drinking status and change in physical activity (PA) from HUNT2 (H2) to HUNT3 (H3).**

|  |  |  | **Model 1** | **Model 2** |
| --- | --- | --- | --- | --- |
|  | **N** | **Deaths** | **HR (95% CI)** | **HR (95% CI)** |
| Abstainer at H2 & H3 - Below PA at H2 & H3 | 221 | 65 | 1.23 (0.86-1.76) | 1.09 (0.76-1.57) |
| Abstainer at H2 & H3 - Below PA at H2 & meeting H3 | 148 | 40 | 1.30 (0.87-1.95) | 1.34 (0.89-2.01) |
| Abstainer at H2 & H3 - Meeting PA at H2 & below H3 | 129 | 40 | 1.41 (0.94-2.11) | 1.41 (0.94-2.12) |
| **Abstainer at H2 & H3 - Meeting PA at H2 & H3** | 250 | 57 | Reference | Reference |
|  |  |  |  |  |
| Abstainer H2, drinker H3 - Below PA at H2 & H3 | 260 | 83 | 1.69 (1.21-2.38) | 1.44 (1.02-2.02) |
| Abstainer H2, drinker H3 - Below PA at H2 & meeting H3 | 192 | 43 | 1.28 (0.86-1.90) | 1.21 (0.81-1.80) |
| Abstainer H2, drinker H3 - Meeting PA at H2 & below H3 | 187 | 47 | 1.81 (1.23-2.67) | 1.58 (1.07-2.32) |
| Abstainer H2, drinker H3 - Meeting PA at H2 & H3 | 311 | 82 | 1.40 (1.00-1.96) | 1.35 (0.96-1.89) |
|  |  |  |  |  |
| Drinker H2, Abstainer H3 - Below PA at H2 & H3 | 23 | 10 | 2.27 (1.16-4.45) | 1.74 (0.88-3.41) |
| Drinker H2, Abstainer H3 - Below PA at H2 & meeting H3 | 19 | 10 | 1.78 (0.91-3.50) | 1.43 (0.73-2.82) |
| Drinker H2, Abstainer H3 - Meeting PA at H2 & below H3 | 18 | 8 | 1.84 (0.88-3.86) | 1.44 (0.68-3.02) |
| Drinker H2, Abstainer H3 - Meeting PA at H2 & H3 | 22 | 9 | 1.05 (0.52-2.11) | 1.00 (0.49-2.01) |
|  |  |  |  |  |
| Drinker H2, Drinker H3 - Below PA at H2 & H3 | 5051 | 878 | 1.58 (1.20-2.06) | 1.30 (0.99-1.70) |
| Drinker H2, Drinker H3 - Below PA at H2 & meeting H3 | 4010 | 621 | 1.40 (1.06-1.84) | 1.26 (0.96-1.66) |
| Drinker H2, Drinker H3 - Meeting PA at H2 & below H3 | 4733 | 669 | 1.51 (1.15-1.98) | 1.27 (0.97-1.68) |
| Drinker H2, Drinker H3 - Meeting PA at H2 & H3 | 9261 | 1249 | 1.25 (0.95-1.63) | 1.19 (0.91-1.56) |

HUNT, the Trøndelag Health Study; HR, hazard ratios; CI, confidence interval; PA, physical activity.

Alcohol consumption categories are based on either abstainers or drinking alcohol. PA below recommendations, and meeting the recommendations (150 minutes of moderate or 75 minutes of vigorous activity or combination).

Model 1 is adjusted for age and sex.

Model 2 is adjusted for age, sex, body mass index, smoking status, hypertension, diabetes, total cholesterol, marital status, and general health status.

**sTable 7. Hazard ratios of all-cause mortality by change in alcohol drinking status from HUNT2 (H2) to HUNT3 (H3), after excluding the first 3 years of follow-up.**

|  | **N** | **Deaths** | **HR (95% CI) *** |
| --- | --- | --- | --- |
| **Abstainer H2 & H3** | 750 | 190 | Reference |
| Abstainer H2, within guidelines H3 | 945 | 239 | 1.21 (1.00-1.46) |
| Abstainer H2, above guidelines H3 | 6 | 0 | - |
|  |  |  |  |
| Within guidelines H2, Abstainer H3 | 82 | 36 | 1.19 (0.83-1.71) |
| Within guidelines H2, within guidelines H3 | 21,610 | 2968 | 1.05 (0.90-1.23) |
| Within guidelines H2, above guidelines H3 | 936 | 118 | 1.28 (1.01-1.62) |
|  |  |  |  |
| Above guidelines H2, Abstainer H3 | 0 | - | - |
| Above guidelines H2, within guidelines H3 | 304 | 49 | 1.42 (1.03-1.96) |
| Above guidelines H2, above guidelines H3 | 220 | 31 | 1.16 (0.80-1.71) |

HR, hazard ratios; CI, confidence interval. Alcohol consumption categories are based on the average weekly intake in grams of pure alcohol: within recommendations (≤140 g/week for men, ≤70 g/week for women) or above recommendations (>140 g/week for men, >70 g/week for women).

^*^ Hazard ratios are adjusted for age, sex, body mass index, smoking status, hypertension, diabetes, total cholesterol, marital status, general health status, and cardiorespiratory fitness.

**sTable 8. Hazard ratios of all-cause mortality by joint association of change in alcohol drinking status and change in CRF from HUNT2 (H2) to HUNT3 (H3), after excluding the first 3 years of follow-up.**

|  |  |  |  |
| --- | --- | --- | --- |
|  | **N** | **Deaths** | **HR (95% CI) *** |
| Abstainer at H2 & H3 |  |  |  |
| Unfit at H2 & H3 | 119 | 50 | 1.77 (1.26-2.49) |
| Unfit at H2 & Fit at H3 | 78 | 19 | 1.21 (0.74-1.98) |
| Fit at H2 & Unfit at H3 | 37 | 7 | 2.43 (1.13-5.23) |
| **Fit at H2 & Fit at H3** | 516 | 114 | Reference |
|  |  |  |  |
| Abstainer H2, within guidelines H3 |  |  |  |
| Unfit at H2 & H3 | 137 | 45 | 1.52 (1.07-2.16) |
| Unfit at H2 & Fit at H3 | 110 | 37 | 1.76 (1.21-2.57) |
| Fit at H2 & Unfit at H3 | 65 | 14 | 2.06 (1.18-3.61) |
| Fit at H2 & Fit at H3 | 633 | 143 | 1.31 (1.03-1.68) |
|  |  |  |  |
| Abstainer H2, above guidelines H3 |  |  |  |
| Unfit at H2 & H3 | 0 | - | - |
| Unfit at H2 & Fit at H3 | 0 | - | - |
| Fit at H2 & Unfit at H3 | 1 | 0 | - |
| Fit at H2 & Fit at H3 | 5 | 0 | - |
|  |  |  |  |
| Within guidelines H2, Abstainer H3 |  |  |  |
| Unfit at H2 & H3 | 16 | 8 | 2.40 (1.17-4.59) |
| Unfit at H2 & Fit at H3 | 8 | 4 | 6.17 (2.27-16.77) |
| Fit at H2 & Unfit at H3 | 2 | 1 | 0.58 (0.08-4.16) |
| Fit at H2 & Fit at H3 | 56 | 23 | 1.14 (0.73-1.79) |
|  |  |  |  |
| Within guidelines H2, within guidelines H3 |  |  |  |
| Unfit at H2 & H3 | 2189 | 424 | 1.66 (1.33-2.07) |
| Unfit at H2 & Fit at H3 | 1945 | 303 | 1.42 (1.14-1.78) |
| Fit at H2 & Unfit at H3 | 1566 | 194 | 1.41 (1.10-1.80) |
| Fit at H2 & Fit at H3 | 15,910 | 2047 | 1.13 (0.93-1.37) |
|  |  |  |  |
| Within guidelines H2, above guidelines H3 - | 63 | 13 | 2.41 (1.35-4.30) |
| Unfit at H2 & H3 | 79 | 15 | 2.19 (1.27-3.76) |
| Unfit at H2 & Fit at H3 | 65 | 7 | 1.36 (0.63-2.93) |
| Fit at H2 & Unfit at H3 | 729 | 83 | 1.31 (0.98-1.75) |
| Fit at H2 & Fit at H3 |  |  |  |

*The table continues on the next page.*

**sTable 8.** Continued.

| Above guidelines H2, Abstainer H3 |  |  |  |
| --- | --- | --- | --- |
| Unfit at H2 & H3 | 0 | - | - |
| Unfit at H2 & Fit at H3 | 0 | - | - |
| Fit at H2 & Unfit at H3 | 0 | - | - |
| Fit at H2 & Fit at H3 | 0 | - | - |
|  |  |  |  |
| Above guidelines H2, within guidelines H3- |  |  |  |
| Unfit at H2 & H3 | 27 | 8 | 2.31 (1.12-4.76) |
| Unfit at H2 & Fit at H3 | 22 | 4 | 2.24 (0.83-6.10) |
| Fit at H2 & Unfit at H3 | 31 | 7 | 4.21 (1.95-9.07) |
| Fit at H2 & Fit at H3 | 224 | 30 | 1.33 (0.89-2.00) |
|  |  |  |  |
| Above guidelines H2, above guidelines H3 |  |  |  |
| Unfit at H2 & H3 | 18 | 5 | 2.50 (1.02-6.15) |
| Unfit at H2 & Fit at H3 | 9 | 2 | 1.59 (0.39-6.43) |
| Fit at H2 & Unfit at H3 | 14 | 3 | 5.93 (1.87-18.80) |
| Fit at H2 & Fit at H3 | 179 | 21 | 1.07 (0.67-1.72) |

HUNT, the Trøndelag Health Study; HR, hazard ratios; CI, confidence interval; CRF, cardiorespiratory fitness. Alcohol consumption categories are based on the average weekly intake in grams of pure alcohol: within recommendations (≤140 g/week for men, ≤70 g/week for women) or above recommendations (>140 g/week for men, >70 g/week for women). CRF: unfit, age-sex specific 20% least fit; or fit, age-sex specific 80% most fit.

* Hazard ratios are adjusted for age, sex, body mass index, smoking status, hypertension, diabetes, total cholesterol, marital status, and general health status.

**sFigure 1. Flow chart of study participants.**

HUNT, the Trøndelag Health Study.


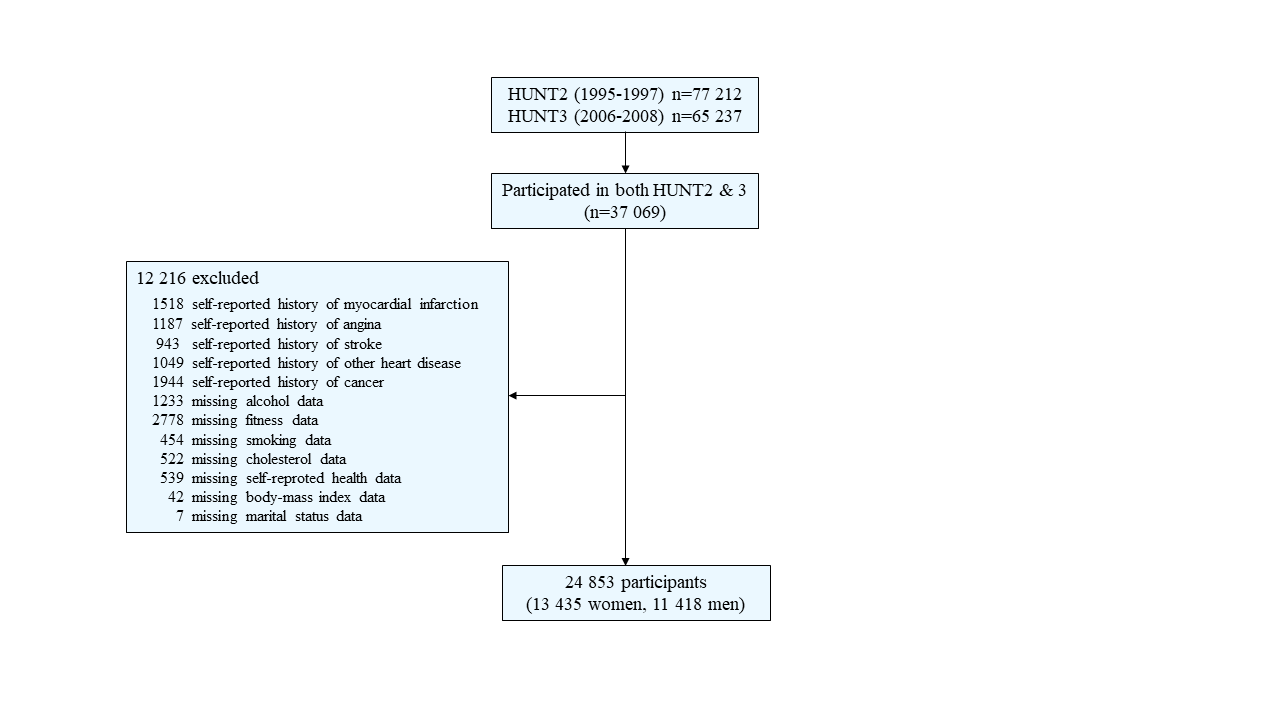


**
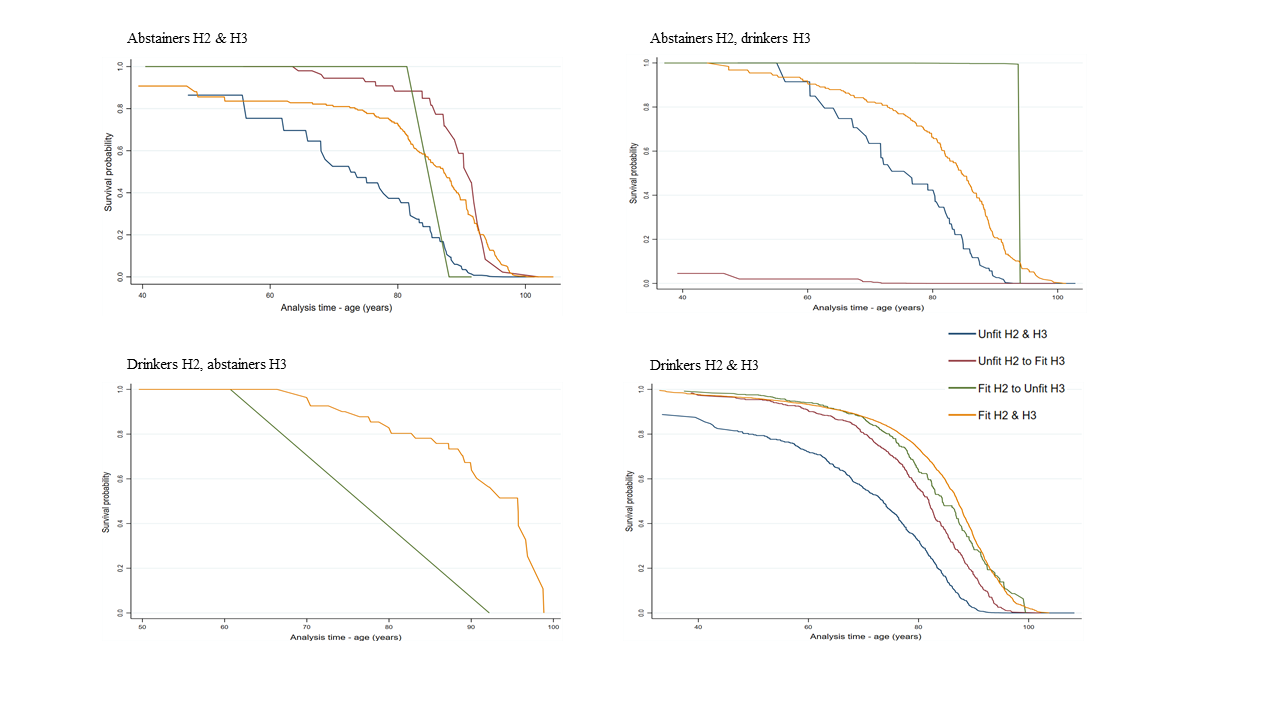
sFigure 2. Survival curves associated with change in cardiorespiratory fitness according to change in alcohol intake**.

H2, HUNT2; H3, HUNT3.

Cardiorespiratory fitness: unfit, age-sex specific 20% least fit; or fit, age-sex specific 80% most fit.
